# Bacterial Proteinaceous Compounds With Multiple Activities Toward Cancers and Microbial Infection

**DOI:** 10.3389/fmicb.2019.01690

**Published:** 2019-08-06

**Authors:** Gisele Rodrigues, Gislaine Greice Oliveira Silva, Danieli Fernanda Buccini, Harry Morales Duque, Simoni Campos Dias, Octávio Luiz Franco

**Affiliations:** ^1^Centro de Análises Proteômicas e Bioquímicas, Programa de Pós-Graduação em Ciências Genômicas e Biotecnologia, Universidade Católica de Brasília, Brasília, Brazil; ^2^S-Inova Biotech, Programa de Pós-Graduação em Biotecnologia, Universidade Católica Dom Bosco, Campo Grande, Brazil; ^3^Pós-Graduação em Biologia Animal, Universidade de Brasilia, Brasília, Brazil

**Keywords:** antimicrobial, anticancer, bacteriocin, protein, peptides

## Abstract

In recent decades, cancer and multidrug resistance have become a worldwide problem, resulting in high morbidity and mortality. Some infectious agents like *Streptococcus pneumoniae*, *Stomatococcus mucilaginous, Staphylococcus* spp.*, E. coli. Klebsiella* spp.*, Pseudomonas aeruginosa, Candida* spp., *Helicobacter pylori*, hepatitis B and C, and human papillomaviruses (HPV) have been associated with the development of cancer. Chemotherapy, radiotherapy and antibiotics are the conventional treatment for cancer and infectious disease. This treatment causes damage in healthy cells and tissues, and usually triggers systemic side-effects, as well as drug resistance. Therefore, the search for new treatments is urgent, in order to improve efficacy and also reduce side-effects. Proteins and peptides originating from bacteria can thus be a promising alternative to conventional treatments used nowadays against cancer and infectious disease. These molecules have demonstrated specific activity against cancer cells and bacterial infection; indeed, proteins and peptides can be considered as future antimicrobial and anticancer drugs. In this context, this review will focus on the desirable characteristics of proteins and peptides from bacterial sources that demonstrated activity against microbial infections and cancer, as well as their efficacy *in vitro* and *in vivo*.

## Introduction

In recent years, global health authorities have had to deal with two significant problems: the alarming number of people suffering from cancer and the rise of antimicrobial resistance (AMR). Cancer is the second most prevalent cause of death worldwide (O’Brien-Simpson et al., 2018; Shoombuatong et al., 2018). According to the World Health Organization (WHO) and the International Agency of Research on Cancer [IARC] (2018) in there were about 18.1 million new cases of cancer and 9.6 million deaths. The estimate for AMR is that 700,000 die annually worldwide, and the annual number of deaths is likely to increase to 10 million by 2050 (Arias and Murray, 2009; World-Health-Organisation [WHO], 2018; Ghosh et al., 2019).

Additionally, 16.1% of newly diagnosed cancers may be attributable to infections [National Cancer Institute – Epidemiology and Genomic Research Program^1^ (accessed March 12, 2019)]. Oncologic patients are more susceptible to infectious complications caused by *Streptococcus pneumoniae*, *Stomatococcus mucilaginous, Staphylococcus* spp.*, E. coli Klebsiella* spp.*, Pseudomonas aeruginosa, Helicobacter pylori*, and *Candida* spp. (Zorina and Styche, 2015; Rolston, 2017). Another concern related to cancer patients are infections caused by viruses such as hepatitis B and C, and human papillomaviruses (HPV) (Vedham et al., 2014; Rolston, 2017).

In fact, patients with a chronic infection induced by *Staphylococcus aureus*, *Klebsiella pneumoniae, Acinetobacter baumanii, Pseudomonas aeruginosa*, and *Enterobacter* species have been shown to have greater susceptibility to cancer development, as a result of their precarious immune system (Felício et al., 2017; Rolston, 2017). At the same time, antibiotics are used to prevent microbial infection in post-cancer surgery, and after chemotherapy or radiotherapy (Thundimadathil, 2012; Gaspar et al., 2013; Felício et al., 2017; Leite et al., 2018; O’Brien-Simpson et al., 2018; Shoombuatong et al., 2018). Conventional cancer treatments do not act on specific targets, such as malignant cells, resulting in severe side effects for patients, and these may contribute to the selection of cells that are resistant to antibiotics and anticancer drugs (Vedham et al., 2014; Zorina and Styche, 2015).

As a result, the development of a new class of molecules with selectivity and specificity against microbial infection and cancer is essential (Gaspar et al., 2013; Felício et al., 2017; Shoombuatong et al., 2018). Bacteria have an arsenal of proteins and peptides with both antibacterial and antitumoral activity, which can be explored in the search for these new compounds (Karpinski and Adamczak, 2018).

Among these promising molecules are toxins, immunotoxins (Jain, 2001; Gorgal et al., 2012), enzymes (Chakrabarty et al., 2014), bacteriocins (which are part of the same group as peptides) and a vast range of proteins (Karpinski and Adamczak, 2018). Antimicrobial peptides (AMPs) with anticancer activity can be classified according to the spectrum of their activity on tumor cells, and they are divided into two main categories: (i) peptides that show potent activity against bacteria and tumor cells, without causing damage to mammalian cells; (ii) peptides that are toxic to cancer cells, bacteria and healthy cells (Hoskin and Ramamoorthy, 2008; Bandala et al., 2013).

Therefore, this review will focus on the desirable characteristics of proteins and peptides originating from bacteria that demonstrated activity against microbial infections and cancer, as well as their efficacy in clinical trials, and will discuss future prospects.

## Dual Activity from Bacterial Proteins and Peptides

**FIGURE 1:**
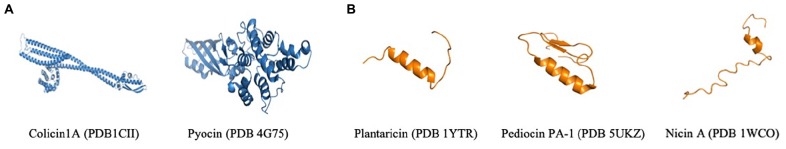
An overview of different structures of bacteriocins, proteins **(A)** and peptides **(B)** with dual anticancer and antimicrobial activity. All of these structures are available on the PDB.

Some proteins and peptides exhibit antimicrobial and anticancer activities. Therefore, bacteria use indirect and direct strategies to compete and survive. Host bacteria can show antimicrobial activity indirectly, by host immune system modulation (Belmadi et al., 2018). Alternatively, host bacteria can act directly by expressing proteins and peptides that are secreted to the extracellular environment and that target other bacteria (Cascales et al., 2007). Likewise, these proteins and peptides present variable structures linked to their activity, and these characteristics make their classification difficult (Daw and Falkiner, 1996; Vasilchenko and Valyshev, 2019). These structural diversities are represented in Figure 1, by toxins like colicin and pycin, and peptides represented by nisin, pediocin and plantaricin (Jain, 2001; Karpinski and Adamczak, 2018). Colicins are formed by three domains, the N-terminal, central and C- terminal, which act in membrane translocation, binding receptor and activity domain, respectively (Cascales et al., 2007). Pyocins are composed of four domains: domain I represents the receptor binding at the N-terminal, domain II has no defined function yet, domain III is responsible for translocation across the outer membrane and domain IV is responsible for DNase activity at the C-terminal end (Michel-Briand and Baysse, 2002). Nisin has two variants, A and Z, and the only difference between them is the change of His^27^ by Asn. They interact with the membrane surface in the C-terminal moiety (Lins et al., 1999). Pediocins interact with the target-cell surface in the N-terminal domain, and the C-terminal domain penetrates the membrane. Indeed, for pedicins, the domain is the major specificity determinant (Fimland et al., 2005). Plantaricin can present three variable forms, with 26-residue peptide and two N-terminal forms containing 23 and 22 residues; these forms result from a 48-residue precursor encoded by the plnA gene. Besides that, the amphiphilic nature of plnA can induced pore formation in cell membrane (Kaur and Kaur, 2015) (Figure 1).

In addition, several strategies could be designed to try to combat antimicrobial infection and carry out cancer therapy using proteins and peptides (Shilova et al., 2018). For example, proteins and peptides can be combined with conventional drugs (Liberio et al., 2013; Felício et al., 2017; Leite et al., 2018). They can be used as a heterologous compound infusion with other proteins or peptides that help in site-directed activity (Kawakami et al., 2006; Leshem and Pastan, 2019). It is possible to coat or conjugate proteins and peptides with polymers, such as polyethylene glycol (PEG) (Kelly et al., 2016). Another strategy is rational design for these molecules, because it then becomes possible to substitute naturally occurring amino acids with unnatural ones (Gordon et al., 2005; Uggerhøj et al., 2015).

**FIGURE 2:**
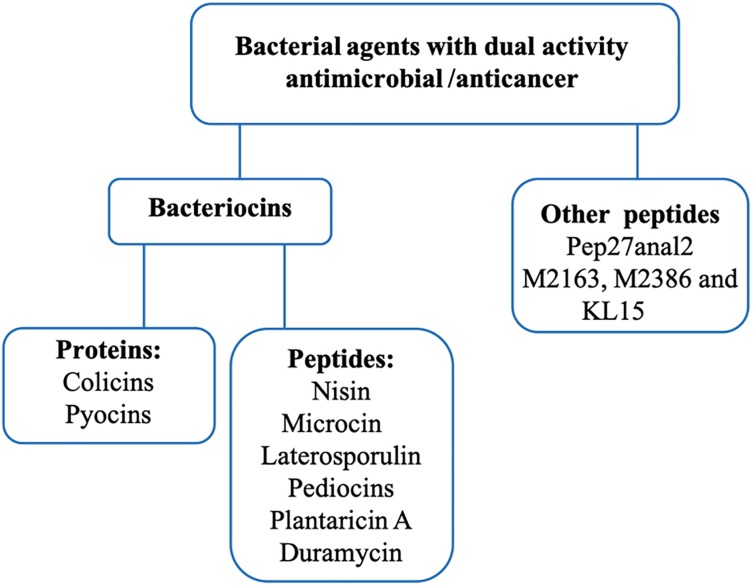
Distribution of described anticancer and antimicrobial proteins and peptides.

In the various ways described above, these molecules have shown different applications and modes of action against antimicrobial infections and cancer. Nevertheless, in this study we focus on bacteriocins that have been previously characterized and/or synthesized with dual activity (Figure 2).

### Bacteriocins

Bacteriocins make up a class of molecules (proteins and peptides) synthesized by ribosomes in several Gram-positive and negative bacteria. In 1925, the first bacteriocin isolated from *E. coli* discovered by Gratia (1925), was identified and later named colicin. Since then, several bacteriocins have been discovered in a variety of bacteria (Kaur and Kaur, 2015). Cationic peptides belonging to the bacteriocin class may be associated with biological functions in bacteria and may assist in the inhibition and elimination of possible competing microorganisms in their natural environment (Nes and Holo, 2000).

As demonstrated above, proteins and peptides have variable structures (Vasilchenko and Valyshev, 2019). Regarding classification, which is a challenge, it is known that 80% of the bacteriocins are of cationic and amphipathic nature, due mainly to the excess of residues of lysine or arginine amino acids (Nes and Holo, 2000; Hammami et al., 2010). These characteristics of bacteriocins may be linked to their efficacy in combating cancer cells, possibly by the interaction of these cationic molecules with the negative surface charge of the cancerous cell membrane (Saito et al., 1979; Baindara et al., 2015). A visible example of such characteristics can be observed in lantipeptides, a class I bacteriocin, which can form pores, resembling cationic antimicrobial peptides (cAMPs) (Smith and Hillman, 2008). In addition, some bacteriocins produced by Gram-positive bacteria can resemble the defensins, AMPs produced by eukaryotic cells, which have membrane-permeabilizing characteristics (Singh et al., 2015).

Another relevant feature of bacteriocins is their low toxicity when in contact with mammalian cells. Due to these characteristics, bacterial strains producing bacteriocins have been implemented in foods as probiotics (Cutter and Siragusa, 1998). Their low toxicity has already been demonstrated in studies, for example, a bacteriocin named laterosporulin did not show hemolysis in the presence of erythrocytes, even at concentrations exceeding the minimum inhibitory concentration (MIC) values (Singh et al., 2015). The same can be observed in studies with penisin, a class IA lantibiotic, which in addition to not being hemolytic showed no cytotoxic activity against mouse macrophages (RAW) (Baindara et al., 2015). These characteristics are important from the point of view of the development of new therapies, since their affinity with tumor cells and low toxicity show that the bacteriocins are excellent candidates for the treatment of cancer, besides presenting antibacterial activities (Chakrabarty et al., 2014; Yang et al., 2014; Kaur and Kaur, 2015; Karpinski and Adamczak, 2018).

**FIGURE 3:**
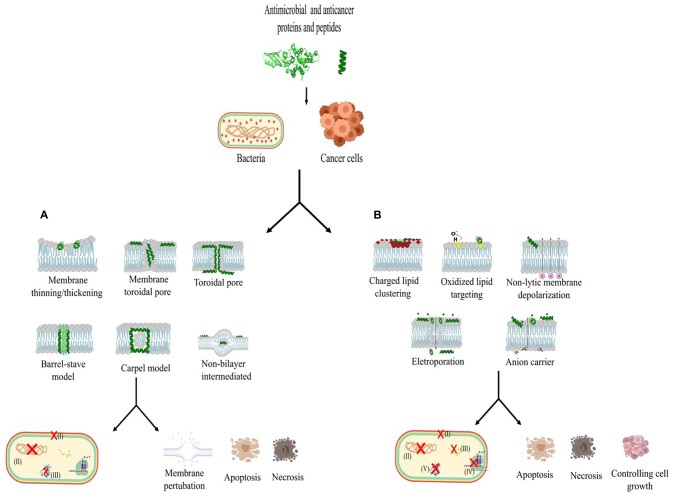
Different mechanisms of action of proteins and peptides with anticancer and antibacterial activity. **(A)** This section represents the protein and peptide interaction with the carpet model, barrel-stave model, toroidal pore, disorder toroidal pore, non-bilayer intermediate and membrane thinning/thickening, which are mechanisms that act in the conformation of the external membrane (I), target the inhibition of phospholipase, lipid II and LPS permeabilization. In the inner membrane, these mechanisms act in DNA synthesis (II) and inhibition of fold protein (III). **(B)** This section represents the action of proteins and peptides interaction the other mechanisms described above, such as anion carrier, charged lipid clustering, electroporation, non-lytic membrane depolarization, and oxidized lipid targeting, act in the destabilization of membrane components (I): in addition, they act in the inner membrane by interrupting DNA (II), RNA(III), and protein syntheses (IV) and (V). All these mechanisms can cause bacterial death and, in cancer cells, can cause disruption of membrane, apoptosis, necrosis, and control of angiogenesis. The model described in **(A)** and **(B)** acting in bacteria and cancer cells.

In this section, we will address the issue of protein and peptide bacteriocins isolated from bacteria. Only those molecules with a molecular mass of 10 kDa or less will be classified as peptides. Among the bacteriocins studied to date, some have been submitted to tests with tumor cells. Table 1 lists the bacteriocins that have been evaluated so far and have demonstrated activity against bacterial and cancer cells (Figure 3). These activities are divided into membrane adsorption (Figure 3A) and conformational change (Figure 3B). Membrane adsorption is represented by carpet model (in which the formation of membrane micellar structures occurs in an area with high peptide densities); barrel-stave model (protein and peptides form a pore in a perpendicular orientation to the membrane surface and interact with the phospholipid acyl chains); toroidal pore model (the contact of protein and peptides with the phospholipid head groups during the pore formations causes expanding membrane curvature); disordered toroidal pore (which promotes the internal curvature of lipid molecules for pore formation with few peptides; this process occurs in randomic form); non-bilayer intermediate (the peptide aggregation on the membrane surface activates the formation of the intermediate bilayer, causing membrane disruption, allowing the peptide to be translocated into the internal leaflet); membrane thinning/thickening (originates with peptides aligned parallel to the surface of the membrane, provoking thinning or thickening due to hydrophilic characteristics associated with the lipid bilayer) (Lohner, 2009; Haney et al., 2010; Nguyen et al., 2011; Gaspar et al., 2013; Gabernet et al., 2016). The conformational charge is represented by anion carrier (this model acts directly on the membrane through lipid isolation or addresses oxidized phospholipids, or even acts as a small anion carrier through the membrane); charge lipid cluster (cationic peptides interact with clustering of anionic lipids in the membrane region, and this interaction allows pore formation); electroporation (occurs subtly, when proteins or peptides bind small anions across the bilayer, inducing the efflux and changes in the membrane potential); non-lytic membrane depolarization (proteins and peptides induce charge modification, cause membrane instability, and allow the proteins and peptides to translocate across the cytoplasmic membrane); and oxidized lipid targeting [stimulating the formation of cellular reactive oxygen species (ROS) causing oxidative cellular damage, developing metabolites that can be mutagenic, cytotoxic and also promote cellular aging and apoptosis] (Gifford et al., 2005; Epand and Epand, 2011; Nguyen et al., 2011; Hasegawa et al., 2012; Gaspar et al., 2013; Gabernet et al., 2016; Alexander et al., 2019).

**TABLE 1:** Anticancer and antimicrobial activity of proteins and peptides from bacterial origins.

**Protein**	**Bacterial origin**	**Size (kDa)**	**Net charge**	**Hydrophobicity < H >**	Antibacterial activity	Cancer cell lines	References
Colicins	*E. coli*	27–80			Gram−	MCF-7, ZR75, BT549, BT474, MDA-MB-231, SKBR3, T47D, HOS, SKUT-1, HS913T, HT29, MRC5.	Karpinski and Adamczak, 2018; Duché and Houot, 2019
Pyocins	*P. aeruginosa*	77–157			Gram+	HFFF, HeLa, AS-II, HepG2, mKS-ATU-7, HCG-27.	Karpinski and Adamczak, 2018

**Peptide**	**Bacterial origin**	**Size (kDa)**	**Net charge**	**Hydrophobicity < H >**	Antibacterial activity	Cancer cell lines	References

Nisin A	*Lactococcus lactis*	3.49	3	0.569	Gram+/Gram−	MCF-7, HepG2, HNSCC, HT29, CaCo-2, SW480.	Kuwano et al., 2005; Maher and McClean, 2006; Joo et al., 2012; Shin et al., 2016; Ahmadi et al., 2017
Nisin Z	*Lactococcus lactis* SIK-83	3.47	3	0.548	Gram+/Gram−	HNSCC, HUVEC.	Kamarajan et al., 2015; Preet et al., 2015
Microcin E492	*Klebsiella pneumoniae*	7.8	−4	0.171	Gram−	Jurkat, RJ2.25 HeLa, CRC cells.	de Lorenzo, 1984; Hetz et al., 2002
Bovicin HC5	*Streptococcus bovis* HC5	2.4	–	–	Gram+/Prevotella bryantii	MCF-7, HepG2	Mantovani et al., 2002; Paiva et al., 2012
Laterosporulin- LS10	*Brevibacillus laterosporus* SKDU10	6.0	2	0.409	*Mycobacterium tuberculosis* and S. aureus	HeLa, H1299, HEK293T, HT1080, MCF-7	Baindara et al., 2015; Singh et al., 2015
Pediocin PA-1	*Pediococcus acidilactici* PAC1	4.6	3	0.343	Gram+	Lung carcinoma (A-549) and CRC (DDL1)	Henderson et al., 1992; Beaulieu, 2005
Pediocin K2a2-3	*Pediococcus acidilactici* K2a2-3	4.1	2	0.360	Gram+	HT29, Hela	Villarante et al., 2011
Pediocin CP2	*P. acidilactici* CP2 MTCC501	4.6	3	0.343	Gram+	Hela, HepG2, MCF-7, Sp2/0-Ag14	Kumar et al., 2011
Plantaricin A	*Lactobacillus plantarum* C11	2.4	6	0.369	Gram+/Gram−	Jurkat, GH_4_, Reh, PC12, N2A.	Nissen-Meyer et al., 1993; Diep et al., 1996; Zhao et al., 2006
m2163	*Lactobacillus casei* ATCC334	2.7	3	0.508	Gram+	SW480	Tsai et al., 2015
m2386	*Lactobacillus casei* ATCC334	2.7	2	0.324	Gram+	SW480	Tsai et al., 2015
KL15	*Lactobacillus casei* ATCC334	1.9	5	0.491	Gram+/Gram−	SW480, CaCo-2	Chen et al., 2015
Duramycin	*Streptoverticillium cinnamoneus*	2.0	1	0.457	Gram+	AsPC-1, Caco-2, Colo320, CT116, JJN3, Lovo, MCF7, MDA-B-231, MIA PaCa-2, MM.1S, U266B1	Iwamoto et al., 2007; Broughton et al., 2016
Pep27	*Streptococcus pneumoniae*	2.8	4	0.040	Gram+/Gram−	Jurkat	Lee et al., 2005; Sung et al., 2007
Pep27anal2	*Streptococcus pneumoniae*	3.3	4	0.527	Gram+/Gram−	Jurkat, HL-60, AML-2, MCF-7, SNU-601	Lee et al., 2005; Sung et al., 2007
p28	*Pseudomonas aeruginosa* PAO1	2.8	−4	0.222	–	MCF-7, HCT-116, UISO-MEL-23, MNE-MB-231, p53wt (Mel-29), U87, LN229	Yamada et al., 2009; Mehta et al., 2011; Bernardes et al., 2013

#### Colicins and Pyocins

There are a small number of proteins classified as bacteriocins, including colicin, pyocin, pesticin, butyricin, and megacin (Konisky, 1982). These proteins are used by bacterial species for intraspecies competition (Di Masi et al., 1973; Chai et al., 1982; Michel-Briand and Baysse, 2002; Spector et al., 2010; Yang et al., 2014). However, only colicin and pyocin have been reported in the literature as presenting dual activity.

Colicins are proteins produced by *E. coli*, and they present a diversity of molecules (E1-3, K, A, L, B, Ia, Ib, V, D, and M) with varied masses between 27 and 80 kDa (Konisky, 1982). The structure of colicin proteins presents three domains; these are the receptor-binding domain responsible for target interaction, the N-terminal domain that mediates interaction with transporters, and the cytotoxic domain that allows antibacterial activity (Vasilchenko and Valyshev, 2019). Colicins act by binding to the outer membrane of integral membrane protein receptors, transporting the colicin to the inner membrane through Tol complex, inducing membrane depolarization, and degradation of DNA, ribosomal RNA, or tRNA (Duché and Houot, 2019) (Figure 3B). Colicins show activity on a wide bacterial range (Duché and Houot, 2019), and they also showed cytotoxic activity on breast carcinoma, fibrosarcoma, leiomyosarcoma, osteosarcoma, and colon carcinoma (Bandala et al., 2013; Karpinski and Adamczak, 2018). In this sense, a study using the different isolates of colicins, E1, E6, E7, K, and M, was evaluated against *E. coli* strains from patients with bacteraemia of urinary tract origin. The colicin isolates were tested in concentrations of 1, 10, and 100 μg/ml. The results showed that colicin E7 was able to kill 87% of the strains tested. The results showed that colicin E7 at 10 μg. ml^–1^ was able to kill 87% of strains tested. The inhibitory activity of combinations of colicins E7, K, M and E1, E6, E7, K effectively inhibited the growth of pathogenic *E. coli* strains (Budič et al., 2011). Furthermore, colicin presents anticancer activity. This activity was elucidated by Chumchalova and Šmarda (2003), in a study that used four colicins (A, E1, U, E3) against 11 cancer cell lines. They detected anticancer activity in most of cell lines tested. Although fibrosarcoma HS913T has demonstrated 50% inhibition with colicin E1, it was also shown to be sensitive to colicin A and U. Only colicin E3 did not exhibit significant inhibition activity against tested cancer cells. Another study demonstrated the efficiency of colicin E3 against murine leukemia P388cells. It tested colicin E3 in final concentrations of 0.4 mg. ml^–1^, 0.8 mg. ml^–1^, 1.6 mg. ml^–1^, and 3.2 mg. ml^–1^ for 0, 24, 48 and 72-h cultivations at 37°C. The results demonstrated the colicin E3 was able to inhibit the proliferation of murine leukemia cells in a time- and dose-dependent manner (Fuska et al., 1979).

Pyocins originate from *P. aeruginosa* (Ghequire and De Mot, 2018); their size varies between 77 and 157 amino acid residues (Ghequire and De Mot, 2014). Three different types of pyocins are described, namely the R, F, and S type. The R-type pyocins connect to the outer membrane receptor and induce the depolarization of the cytoplasmic membrane in relation to pore formation (Figure 3A). S-type or colicin-like pyocins show endonuclease activity when placed at the C-terminal end, and this activity induces cell death by interrupting DNA, RNA and protein syntheses (Michel-Briand and Baysse, 2002) (Figure 3B). These proteins act on Gram-negative bacteria (McCaughey et al., 2016), and demonstrated activity against embryonal ovary carcinoma, human hepatocellular carcinoma, and cervical adenocarcinoma (Karpinski and Adamczak, 2018).

Thus, Ling et al. (2010) studied the pyocin S5 in different concentrations (3.5, 14, 56, and 225 μg. ml^–1^) against seven clinical *P. aeruginosa* isolates (DWW3, InA, InB, In3, In4, In7, and In8). Their results indicated that isolate DWW3 is most sensitive in an inhibitory concentration of 12.6l μg.ml^–1^, and the other isolates were killed in a concentration of 225 μg. ml^–1^ isolates (Ling et al., 2010). Additionally, R-type pyocins were tested in a lethal mouse peritonitis model. The researchers infected female mice (Charles River Laboratories) with 0.5. ml^–1^ of inoculum of *P. aeruginosa* strain 13s per mouse, in concentrations of 10^6^ to 10^7^ CFU. ml^–1^. The treatment with 0.1. ml^–1^ of pyocins was administered intraperitoneally (i.p.) and intravenously (i.v.). At 24 and 48 h after treatment inoculation, blood and spleen samples were evaluated. The result showed that i.p. and i.v. dose responses were similar, and pyocin killed approximately 99.99% of the bacteria in the blood and spleen samples. This study suggested that R-type pyocins could be an effective therapy (Scholl and Martin, 2008).

A different test using purified pyocin S2 and partially purified pyocin from *P. aeruginosa* 42A was evaluated against human tumor cell lines, HepG2 and Im9, and the normal human cell line HFFF (Human Fetal Foreskin Fibroblast). Pyocin S2 and partially purified pyocin were tested at concentrations of 6.25, 12.25, 25, and 50 U. ml^–1^, for 5 days. The results showed cell line Im9 was more sensitive than HepG2, and maximum growth inhibition of 80% was observed at the maximum pyocin concentration of 50 U. ml^–1^ after 5 days (Abdi-Ali et al., 2004). According to the examples cited above, the colicins and pyocins demonstrated efficacious antimicrobial and anticancer activity.

#### Nisin

Nisin can be secreted by *Lactococcus lactis* subspecies lactis and has a low molecular mass of 3.49 kDa with 34 amino acid residues. This polycyclic peptide belongs to a class of lantibiotics, which have unusual amino acids such as lanthionines (Moll et al., 1997; de Arauz et al., 2009; Shin et al., 2016). Nisin exhibits stability at different temperatures and has potent activity against different bacteria, including pathogenic strains, acting primarily on Gram-positive bacteria (Jack et al., 1995; Severina et al., 1998). In view of this, nisins are classified as broad-spectrum peptides, presenting activity in different classes of bacteria (Shin et al., 2016). In addition, nisins share some similarities with pore-forming AMPs, such as positive net charge and amphipathicity. These peptides may exhibit different mechanisms of activity that may involve interaction with membranes facilitated by lipid II binding (Christ et al., 2007). This can lead to the formation of pores in the membrane (Moll et al., 1997) (Figure 3). Due to its low toxicity, this molecule has been used for a long time in food preservation (Gharsallaoui et al., 2016). In addition to its antibacterial activity, nisin and its natural variants have been shown to be effective in combating cancerous cells (Joo et al., 2012; Kamarajan et al., 2015).

In this sense, several studies have been carried out to demonstrate the efficiency of nisin against microorganisms. One of these studies purified nisin Z, which exhibits antimicrobial activity against *S. aureus* and *E. coli*. However, the test using nisin Z and 100 mM NaCl demonstrated activity just for *S. aureus* (Kuwano et al., 2005). Another study described the nisin activity against cariogenic microorganisms (*Streptococcus* spp., *Lactobacillus* spp., *Actinomyces* spp.). According to the researchers, electron microscopy showed that nisin exerted bactericidal activity by forming small pores on the surface of cells (Tong et al., 2010) (Figure 3A).

In addition, we described studies using nisin with anticancer activity. Joo et al. (2012) demonstrated that nisin decreases head and neck squamous cell carcinoma (HNSCC) tumorigenesis *in vitro* and *in vivo* by inducing increased cell apoptosis and decreased cell proliferation. For the *in vitro* test, the researchers used a concentration of 5, 10, 20, 40, and 80 μg.ml^–1^ nisin against three different HNSCC cell lines, and after 24 h of treatment they observed increased levels of DNA fragmentation or apoptosis. *In vivo* tests were evaluated with a 150 mg/kg dose of nisin administered over the course of 3 weeks. They observed that mice that had received nisin treatment exhibited statistically significant reduced tumor volumes compared with control. These effects are associated with the activation of CHAC1, broadening calcium influxes and inducing cell cycle arrest (Joo et al., 2012).

Another group also tested the efficiency of nisin Z for the treatment of HNSCC *in vitro* and *in vivo.* They used nisin Z at different concentrations (0, 100, 400, and 800 μg.ml^–1^) against normal-human umbilical vein endothelial cells (HUVECs) in an *in vitro* test. In *in vivo* tests, they used an oral cancer floor-of-mouth mouse model. The treatment started after confirmation that tumors were established, using a control group that was given water (equal volume/control), and the treatment groups were treated with nisin, at two different concentrations (400 and 800 mg/kg per day) by oral gavage for 3 weeks. The researchers observed that the *in vitro* test demonstrated notably increasing levels of apoptosis when compared to cells treated with control medium. Furthermore, the concentrations of nisin tested *in vivo* significantly decreased the tumor volumes (13.5 and 88.5 mm^3^ for nisin ZP 800 mg/kg, nisin ZP 400 mg/kg, respectively) compared to controls (232.8 mm^3^). The authors concluded that nisin is an alternative therapy for HNSCC, exhibiting HNSCC cell apoptosis, suppressing HNSCC cell proliferation, inhibiting angiogenesis, HNSCC and tumorigenesis *in vivo* (Kamarajan et al., 2015). Different studies demonstrated the capacity of nisin to reduce, control and increase the apoptosis of distinct types of tumor cells (Preet et al., 2015; Ahmadi et al., 2017).

In addition, bovicin HC5 is a peptide with a strong similarity to the structure and function of nisin. This peptide can cause cell membrane disruption through pore formation and by modifying cellular potassium efflux (Figure 3A). It is a lantibiotic secreted by *Streptococcus bovis* HC5 and presents about 2.4 kDa of molecular mass. It can be considered as an AMP with a broad spectrum of activity on Gram-positive and negative bacteria. It is considered a stable molecule at high temperatures and low pH, but it may undergo loss of activity in the presence of pronase E and trypsins (Mantovani et al., 2002). Bovicin HC5 has already demonstrated anticancer activity against human adenocarcinoma cells (MCF-7) and hepatocellular carcinoma (HepG2) (Paiva et al., 2012).

#### Microcin

Bacteria belonging to the Enterobacteriaceae family are responsible for the production of microcin. These peptides can have a molecular mass up to 10 kDa, and they present activity on different strains of pathogenic bacteria, such as *Salmonella, Enterobacter, Klebsiella, Escherichia*, and *Citrobacter* (de Lorenzo, 1984). The microcins are classified in two classes: class I is represented by peptides with molecular masses <5 kDa, being codified by cluster genes located either on plasmids or on the chromosome, like microcins B17, C (or C7, C51 or C7/C51), and J25.6. Class II is represented by higher molecular mass peptides ranging from 5- to 10-kDa. Additionally, they are subdivided into two subclasses, IIa and IIb. Class IIa presents three plasmid-encoded peptides, microcins L, V, 6 and N. They do not have post-translational modifications and can present two, one, and no disulfide bond(s), respectively. Class IIb is described as linear microcins with post-translational modification represented by microcins E492, M, H47, and presumably I47 and G492 (Rebuffat, 2013). The microcin act taking advantage of iron-siderophore receptors (FepA, Cir, Fiu). After receptor interaction, microcin was translocated though the TonB-ExbB-ExbD inner-membrane protein complex (Morin et al., 2011; Rebuffat, 2013). Thus, microcin induces depolarization of the cytoplasmic membrane, reaching a specific molecular target (de Lorenzo, 1984; de Lorenzo and Pugsley, 1985; Lagos et al., 1993; Hetz et al., 2002; Morin et al., 2011; Rebuffat, 2013) (Figure 3A).

The efficiency of microcin was studied by Yu et al. (2018). They tested microcin (MccJ25) against the ETEC K88 (serotype O149:K91, K88ac) strain, which is a pathogen related with human infants and neonatal diarrhea. They used MccJ25 in 0.125 to 256 μg/mL concentrations. The results demonstrated the efficacy of MccJ25 at a concentration of 0.25 μg. ml^–1^ against ETEC K88. They observed that MccJ25 was not cytotoxic, and that is was also able to protect the intestine against ETEC K88-induced damage and inflammatory response. According to the authors, MccJ25 can be used as a novel prophylactic agent to reduce pathogenic infection in animals, food or humans.

Furthermore, the efficacy of microcin with *in vitro* tumor cells was described by Hetz et al. (2002). They studied microcin E492 produced by *Klebsiella pneumoniae* strain RYC492 with 7,887 Da. This peptide was tested against some human tumor cells such as Jurkat, RJ2.25, colorectal carcinoma (CRC) and HeLa to observe the capacity to induce apoptosis. The authors tested microcin E492 at different concentrations of 5, 10, and 20 μg/ml. The result demonstrated that at a concentration of 5 μg. ml^–1^ microcin induced tumor cells to apoptosis, and at concentrations of 10 and 20 μg. ml^–1^ necrosis was observed (Hetz et al., 2002).

#### Laterosporulins

Laterosporulins (LS) are peptides with low molecular masses (approximately 5.6 kDa) that present a strong similarity to defensins (Singh et al., 2015). This class of peptides exhibits amphiphilic helical structure, which permits laterosporulin to insert itself into the membrane of the target cell, inducing depolarization and death (Kaur and Kaur, 2015) (Figure 3A). These peptides were isolated from different strains, namely GI-9 and SKDU10, from *Brevibacillus* sp. and have showed potent antibacterial activity against several pathogens (Singh et al., 2015). One example is laterosporulin 10 (LS10) isolated from *Brevibacillus* SKDU10, with 6 kDa, which was tested at different concentrations of 4, 8, and 20 μM against *Staphylococcus aureus* and *Mycobacterium tuberculosis* (MtbH37Rv). The result showed that LS10 inhibited the growth of *S. aureus* with LD50 of 4.0 μM and *M. tuberculosis* (Mtb H37Rv) with LD50 of 0.5 μM. In addition, microscopic studies demonstrated that LS10 acts on *S. aureus* cell membrane and the Mtb H37Rv strain by disrupting cellular metabolic homeostasis. LS10 was able to alter the membrane of the Mtb H37Rv strain, which has a thick lipid layer (Baindara et al., 2016). Moreover, regarding LS10 antitumor activity, this peptide was tested in the concentrations 1–20 μM against HeLa, H1299, HEK293T, HT1080, MCF-7, and RWPE-1 cells. The results demonstrated that LS10 showed activity against diverse cancer cells like MCF-7, HEK293T, HT1080, HeLa, and H1299 in low concentrations (10 μM), but failed against RWPE-1 cells. Moreover, the LS10 at a concentration of 2.5 μM induced apoptosis (Baindara et al., 2018).

#### Pediocins

Pediocins originate from bacteria that produce lactic acid, mainly species of *Pediococcus* (Kumar et al., 2011). A variety of pediocins have been identified so far, namely, pediocin CP-2, F, K1, AcH, AcM SJ-1, and L50, some of which are cited in an extensive review by Porto et al. (2017). They can be considered as small plasmid-encoded cationic AMPs (>5 kDa), with high stability at a variety of temperatures and pHs. However, they may undergo actions of different proteolytic enzymes such as trypsin, α-trypsin, pepsin, papain, and proteases (Kumar et al., 2011). Their N-terminal region contains the conserved Y-G-N-G-V/L “pediocin box” motif and two conserved cysteine residues that are joined by a disulfide bridge, which forms a three-stranded antiparallel beta-sheet (Papagianni and Anastasiadou, 2009). Pediocins contain a conserved N-terminal region folded by disulfide bonds, and this domain mediates binding of class IIa bacteriocins to the target cell membrane. In contrast, the C-terminal region forms a hairpin that is able to penetrate the target cell membrane hydrophobic region, thereby mediating leakage through the membrane (Fimland et al., 2005; Drider et al., 2006) (Figure 3A). Pediocins have showed antimicrobial activity. According to Bédard et al. (2018), they synthesized pediocin PA-1 and demonstrated that it is a potent inhibitor of *Listeria monocytogenes* (MIC = 6.8 nM), similar to that produced naturally by *Pediococcus acidilactici*. Pediocin PA-1 was also tested against different strains of *Carnobacterium divergens* ATCC 35677 (MIC = 1.9 nM), *Leuconostoc mesenteroides* ATCC 23386 (MIC = 1.9 nM), *Listeria seeligeri* ATCC 35967 MIC = 4.7 nM), *Clostridium perfringens* AAC 1–222 (MIC = 37.8 nM), *Clostridium perfringens* AAC 1–223 (MIC = 75.7 nM), *Listeria murrayi* ATCC 25401 (MIC = 151.4 nM), and *Lactobacillus plantarum* ATCC 8014 (MIC = 605.5 nM). Studies have shown the antitumor activity of some pediocins, as exemplified by PA-1, a pediocin produced by *P. acidilactici* PAC1. In the presence of human lung carcinoma cells and colorectal adenocarcinoma, PA-1 inhibited the growth of these cells. Pediocin PA-1 isolated from *P. acidilactici* K2a2-3 has also been shown to be cytotoxic to human colon adenocarcinoma HT29 and HeLa cells, but the mechanism of cytotoxicity has not been studied (Villarante et al., 2011). As also observed, pediocin CP2 produced by *P. acidilactici* CP2 MTCC501 has antitumor activity on HEPg2, HeLa and MCF-7 human cancer cells (Kumar et al., 2011).

#### Plantaricin

The peptide plantaracin (Pln) is produced by different strains of *Lactobacillus plantarum* (C11, WCFS1, V90), showing low molecular mass (∼2.4 kDa) (Diep et al., 1996). The amphiphilic nature of the Pln peptide (class IIb), could facilitate the formation of membrane channels (Nissen-Meyer et al., 1993) (Figure 3A). In fact, Pln has already been shown to permeate eukaryotic cells, but demonstrates affinity to negatively charged membranes and exhibits strong interaction with glycolate membrane proteins (Sand et al., 2013). Some works have demonstrated a broad-spectrum activity of these peptides on different bacterial strains (Nissen-Meyer et al., 1993; Diep et al., 1996). A recent study demonstrated the antimicrobial activity of plantaricins (Pln) A, E, F, J, and K against *Staphylococcus epidermidis*. The plantaricins alone were tested at a concentration of 0.097 to 50 μM, and the plantaricins in association with the antibiotics were tested at concentrations12.5 and 6.25 μM. The results showed that *S. epidermidis* was more susceptible to PlnEF than PlnJK, with MIC 12.5 and 25 μM, respectively. PlnE, F, J and K inhibited bacterial growth, and PlnEF and PlnJK, at 25 and 50 μM, caused rapid bacterial lysis (data not shown). In addition, PlnA (50 μM) alone repressed bacterial growth. Pln in combination with low concentrations of antibiotics displayed antimicrobial activity against *S. epidermidis*. According to results, Pln in combination with antibiotics in low concentration was efficient against *S. epidermidis* and exhibited strong potential to treat infections (Selegård et al., 2019). Pln also demonstrated activity on cancer cells (Zhao et al., 2006; Sand et al., 2010, 2013). This activity was shown in a study by Sand et al. (2010), in which they studied the effect of synthesized PlnA against normal human B and T cells, Reh cells (from human B cell leukemia), and Jurkat cells (from human T cell leukemia). The cell types were tested at a concentration of 10–100 μM PlnA. The results showed that all cells were affected by PlnA, but at low concentrations (10 μM) this did not demonstrate a strong effect. The mechanism of action was seen to be membrane permeabilization, leading to apoptosis along with necrosis.

#### Duramycin

Duramycin is a type of lantibiotic produced by streptomycetes. This tetracyclic peptide is synthesized by ribosomes and exhibits post-translational changes, as well as possessing antimicrobial activity. It consists of 19 amino acid residues corresponding to a molecular mass of ∼2 kDa (Phoenix et al., 2015; Broughton et al., 2016). The post-translational changes undergone by duramycin, such as the enzymatic addition of three thioether bonds, besides increasing the proteolytic stability of this molecule, also provide selectivity and binding to phosphatidylethanolamine (PE) present on the membrane of various cell types, including Gram-positive and negative bacteria (Iwamoto et al., 2007). The interaction of duramycin with target cells can trigger plasmatic membrane imbalance, affecting membrane integrity and influencing the ion transportation mediated by pore formation on the surface of the cell membrane (Sheth et al., 1992; Oliynyk et al., 2010) (Figure 3A).

Other effects of duramycin on the plasma membrane have been shown, such as an inhibitory effect on plasma membrane ATPase activity (Nakamura and Racker, 1984), an increase in cell membrane permeability, and inhibition of Na^+^-K^+^-ATPase in the cellular plasma membranes of Ehrlich ascites tumor cells (Nakamura and Racker, 1984). Due to these characteristics, the antineoplastic capacity of duramycin has been studied. Since the membrane surface of some cancer cells is positive for PE, the effectiveness of duramycin on these cells was visible, decreasing the proliferation of tumor cells and inducing apoptosis. In accordance with a study conducted by Broughton et al. (2016), it was shown that about 11 cancer cell lines (AsPC-1, Caco-2, Colo320, HCT116, JJN3, Lovo, MCF-7, MDA-MB-231, MIA PaCa-2, MM. 1S, and U266B1) expressed PE on the surface. Furthermore, cell death from necrosis in these cancer cells and the release of Ca 2^+^ calcium ions were identified, depending on the time of exposure as well as the concentration of duramycin. Other findings such as morphological changes and influx of iodide have also been reported (Broughton et al., 2016).

### Other Peptides

#### Pep27anal2

Pep27anal2 contains 27 amino acid residues and has a molecular mass of 3.3 kDa. This peptide is an analog of pep27, produced by *Streptococcus pneumoniae* (Sung et al., 2007). Therefore, pep27 showed MIC 12.5 for Gram-positive and Gram-negative bacteria without a hemolytic effect on human erythrocytes (Sung et al., 2007). Pep27anal2 has a higher number of hydrophobic residues compared to the native peptide pep27. This hydrophobic characteristic of pep27anal2 may be related to the interaction with cell membranes and possibly the anticancer activity that this peptide has demonstrated (Figure 3A) (Lee et al., 2005). It demonstrated activity against leukemia cancer cells (Jurkat, HL-60, AML-2), breast cancer (MCF-7) and gastric cancer (SNU-601). Besides the ability to permeate cancer cells, data indicate that the mechanism responsible for cytotoxicity in neoplastic cells arises from the induction of apoptosis of caspase-free and cytochrome-C. In addition, electron microscopy revealed that pep27anal2 induced the morphological features of apoptosis in Jurkat cells, and showed cytoplasmic condensation, cell shrinkage, loss of plasma membrane microvilli, condensed or fragmented nuclei, and the formation of membrane vesicles (Lee et al., 2005). Considering its potent activity against cancer cells, pep27anal2 is a potential candidate for antineoplastic therapy (Lee et al., 2005).

#### M2163, M2386, and KL15

Based on genomic analyses of *Lactobacillus casei* ATCC334, some DNA sequences responsible for the expression of the antimicrobial peptides m2163 and m2386 were identified (Tsai et al., 2015). These peptides demonstrated activity on different lactobacillus strains as well as species of *Listeria* sp. Furthermore, m2163 and m2386 showed effective activity on SW480 cancer cells, acting on the cell membrane and then penetrating the cell cytoplasm to induce apoptosis (Figure 3B) (Tsai et al., 2015). These bacteriocins, m2163 and m2386, were the sources of inspiration for the development of the KL15 antimicrobial peptide through *in silico* modifications in their sequences. KL15, besides having potent antibacterial activity on pathogenic bacteria such as *Enterococcus, Staphylococcus, Bacillus, Escherichia*, and *Listeria*, presented anticancer activity against SW480 and CaCo-2 human adenocarcinoma cells (Chen et al., 2015). Moreover, KL15 (50 μg. mL^–1^) has been shown to be able to permeate the membranes of SW480 cells, resulting in the formation of porous structures, resulting in necrotic cell death. However, the 150 μg. mL^–1^ dose of KL15 showed cytotoxicity on human normal mammary epithelial cells H184B5F5/M10 (Chen et al., 2015).

## Final Remarks

As mentioned above, cancer and chronic infections are the predominant causes of death worldwide. The conventional treatment for these problems generates resistance against multiple drugs. Moreover, conventional treatments are not efficient and effective, inducing serious side effects in patients (Liu et al., 2015; Felício et al., 2017; Leite et al., 2018).

Thus, a new class of molecules needs to be developed and used to provide a more targeted therapy, by exploiting more specific interactions between the drugs and their targets (Gaspar et al., 2013; Felício et al., 2017; Wang et al., 2017; Leite et al., 2018; Shoombuatong et al., 2018). To this end, bacteria have an arsenal of proteins and peptides with both antibacterial and antitumoral activity, which can be explored in the search for these new compounds (Karpinski and Adamczak, 2018).

Various approaches have been discussed here, demonstrating the properties of proteins and peptides derived from bacteria as an alternative strategy for cancer treatment. Among the proteins and peptides that act against bacterial and cancer cells, several stand out. Colicins act on a wide range of bacteria (Duché and Houot, 2019), and have also demonstrated activity against breast carcinoma, fibrosarcoma, leiomyosarcoma, osteosarcoma, and colon carcinoma (Karpinski and Adamczak, 2018). Another promising group is the nisins, which act on Gram-positive bacteria (Jack et al., 1995; Severina et al., 1998), and some nisin variations have demonstrated activity against Gram-negative bacteria (Kuwano et al., 2005), besides potent activity on HNSCC, reducing tumorigenesis (Joo et al., 2012).

Proteins and peptides have been studied for some years, but recently the number of publications and *in vivo* tests have increased. This has led to the rising number of proteins and peptides approved for use in medical practice. In addition, it is to be expected that in upcoming years these molecules may replace conventional treatments. Thus, it is necessary to improve some properties of these molecules in order to decrease or eliminate cytotoxic effects and increase the specificity of the targeting. Besides that, to expand the use of proteins and peptides it will be important to combine these molecules with conventional drugs. This can reduce costs per treatment, besides decreasing the resistance problem. Therefore, proteins and peptides derived from bacteria with dual activity are an important alternative to current treatments against infections and cancer, reducing side effects and curbing the rise of resistant bacteria.

## Author Contributions

All authors listed have made a substantial, direct and intellectual contribution to the work, and approved it for publication.

## Conflict of Interest Statement

The authors declare that the research was conducted in the absence of any commercial or financial relationships that could be construed as a potential conflict of interest.

## Footnotes

1 https://epi.grants.cancer.gov/infectious-agents/#web
